# Rapidly Increasing Serum 25(OH)D Boosts the Immune System, against Infections—Sepsis and COVID-19

**DOI:** 10.3390/nu14142997

**Published:** 2022-07-21

**Authors:** Sunil J. Wimalawansa

**Affiliations:** Endocrinology & Nutrition, Department of Medicine, Cardiometabolic & Endocrine Institute, North Brunswick, NJ 08873, USA; suniljw@hotmail.com

**Keywords:** calcifediol, coronavirus, endocrine system, hypovitaminosis D, immune system, human nutrition, SARS-CoV-2, vitamin D1

## Abstract

Vitamin D deficiency is a global public health problem, a pandemic that commonly affects the elderly and those with comorbidities such as obesity, diabetes, hypertension, respiratory disorders, recurrent infections, immune deficiency, and malignancies, as well as ethnic minorities living in temperate countries. The same groups were worst affected by COVID-19. Since vitamin D deficiency weakens the immune system, it increases the risk of infections, complications, and deaths, such as from sepsis and COVID-19. Deficiency can be remedied cost-effectively through targeted food fortification, supplementation, and/or daily safe sun exposure. Its endocrine functions are limited to mineral metabolism, musculoskeletal systems, specific cell membrane interactions, and parathyroid gland functions. Except for the rapid, endocrine, and cell membrane-based non-genomic functions, all other biological and physiological activities of vitamin D depend on the adequate intracellular synthesis of 1,25(OH)_2_D (calcitriol) in peripheral target cells via the genome. Calcitriol mediates autocrine (intracrine) and paracrine signalling in immune cells, which provides broader, protective immune functions crucial to overcoming infections. The synthesis of 1,25(OH)_2_D (calcitriol) in peripheral target cells is dependent on diffusion and endocytosis of D_3_ and 25(OH)D from the circulation into them, which requires maintenance of serum 25(OH)D concentration above 50 ng/mL. Therefore, in acute infections such as sepsis and respiratory infections like COVID-19, it is necessary to rapidly provide its precursors, D_3_ and 25(OH)D, through the circulation to generate adequate intracellular calcitriol. Immune defence is one of the crucial non-hormonal functions of vitamin D. A single oral (bolus) dose or divided upfront loading doses between 100,000 and 500,000 IU, using 50,000 IU vitamin D_3_ increase the serum 25(OH)D concentrations to a therapeutic level of above 50 ng/mL that lasts between two to three months. This takes three to five days to raise serum 25(OH)D. In contrast, a single oral dose of calcifediol (0.014 mg/kg body weight) can generate the needed 25(OH)D concentration within four hours. Considering both D_3_ and 25(OH)D enter immune cells for generating calcitriol, using the combination of D_3_ (medium-term) and calcifediol (immediate) is cost-effective and leads to the best clinical outcome. To maximise protection against infections, particularly to reduce COVID-19-associated complications and deaths, healthcare workers should advise patients on safe sun exposure, adequate vitamin D supplementation and balanced diets containing zinc, magnesium, and other micronutrients to support the immune system. Meanwhile, governments, the World Health Organisation, the Centers for Disease Control, and governments should consider similar recommendations to physicians and the public, change the outdated vitamin D and other micronutrient recommendations directed to their population, and organise targetted food fortification programs for the vulnerable groups. This article discusses a rational approach to maintaining a sustained serum 25(OH)D concentration above 50 ng/mL, necessary to attain a robust immune system for overcoming infections. Such would cost-effectively improve the population’s health and reduce healthcare costs. It also describes three cost-effective, straightforward protocols for achieving and sustaining therapeutic serum 25(OH)D concentrations above 50 ng/mL (>125 nmol/L) to keep the population healthy, reduce absenteeism, improve productivity, and lower healthcare costs.

## 1. Introduction

The endocrine functions of vitamin D encompass the musculoskeletal system, membrane—vitamin D- interactions mediations actions, and vitamin D−ionised calcium-dependent parathyroid gland functions, calcium and phosphate metabolism including skeletal mineralisation and gastrointestinal and renal tubular calcium absorption. In contrast, peripheral target cell signalling and functions depend on the diffusion of sufficient quantities of precursors of calcitriol—vitamin D_3_ and 25(OH)D from the circulation into immune cells. Most vitamin D-dependent peripheral target cell functions, including intracrine/autocrine and paracrine signalling, are determined by the adequate intracellularly generation of calcitriol from 25(OH)D via its 1α-hydroxylation.

Low circulating concentrations of D_3_ and 25(OH)D hinder their entry into immune cells and, thus, generation of calcitriol. Therefore, vitamin D deficiency (i.e., low vitamin D and 25(OH)D in the circulation) impairs the beneficial effects of vitamin D from peripheral target cells, such as immune cell functions. Consequently, hypovitaminosis D increases the vulnerability to infections and worsens chronic diseases and the severity of illnesses, leading to higher complications and premature deaths. Despite vitamin D supplements being widely available and economical to use and the availability of sunlight worldwide, vitamin D inadequacy is highly prevalent. Chronic vitamin D deficiency significantly increases ill-health, reduces productivity, and escalates healthcare costs [1,2].

Sometime during the year, over half of the world’s population is subjected to vitamin D deficiency, thus increasing their vulnerability to infections, and worsening chronic diseases [2]. Because hypovitaminosis is D-associated adverse effects affecting multiple systems, we estimated that it contributes to a fourth of the overall healthcare costs. In contrast, the population’s vitamin D sufficiency improves the overall health of individuals and the population and significantly lower healthcare costs. In minority ethnic groups and vulnerable populations, vitamin D status can be improved with the targeted, adequate fortification of staple foods with cholecalciferol (D_3_) (and other micronutrients).

However, the typical doses recommended for people and food fortification by governments, their affiliated entities, and medical societies are too small to ensure adequate population repletion [1,3]. In 70 kg adults ingesting vitamin D_3_ doses of 5000 IU/day (0.125 mg/day) could achieve a plateau of serum 25(OH)D above 50 ng/mL but will take a few months [4,5]. Therefore, it is ineffective in emergencies. However, this delay can be reduced to a few days by administering a larger upfront loading dose or one mg of partially activated vitamin D, calcifediol [25(OH)D], as described below [6].

Since most people worldwide are vitamin D deficient sometime during the year and those with hypovitaminosis D, have a higher risk for infections, in emergencies, and acute exacerbation of chronic diseases, the upfront loading of vitamin D can effectively and rapidly raise serum 25(OH)D concentrations. Very high doses of vitamin D have been successfully used under specialists’ guidance for conditions such as migraine and cluster headaches, psoriasis, and autoimmune diseases like multiple sclerosis, rheumatoid arthritis, inflammatory bowel diseases, connective tissue disorders, etc. [4,6]. These are discussed below in Section 4.2.

### 1.1. Hormonal Actions of Calcitriol—Vitamin D Metabolism

The hormonal form of calcitriol is generated in proximal renal tubular cells by renal 1α-hydroxylase (CYP27B1), which hydroxylates 25(OH)D to the most bioactive form, 1,25(OH)_2_D (calcitriol), and transport via the circulation bound to vitamin D binding protein (VDBP). This conversion is tightly regulated by three hormones—1α,25(OH)_2_D, FGF23, the parathyroid hormone (PTH), and ionised calcium. The expression of renal CYP27B1 mRNA is upregulated by PTH and activated through the adenyl cyclase system.

Through direct (genomic) and indirect (non-genomic) actions (see Section 1.3 for details), circulatory calcitriol inhibits parathyroid hormone (PTH) synthesis in chief cells in the parathyroid gland. Calcitriol-mediated enhanced intestinal calcium absorption and the resultant elevated circulating ionised calcium indirectly reduces PTH secretion. Whilst calcitriol is a negative regulator of PTH—transcription of the PTH gene (that takes several hours) and resultant PTH stimulates renal cell calcitriol synthesis. This circulatory, hormonal form of calcitriol is essential for maintaining calcium and phosphate homeostasis, physiological functions of the musculoskeletal system, parathyroid cell functions, and non-genomic cell-membrane interactions [7].

Vitamin D metabolism is regulated by PTH and fibroblast growth factor-23 (FGF-23), which regulate calcium and phosphate homeostasis tightly. The parathyroid gland secretes PTH in response to ionised calcium concentration via the calcium-sensing receptors (calcium homeostasis). Low calcitriol stimulates the expression of CYP27B1 and increases intracellular calcitriol in renal cells. Meanwhile, high concentrations of circulatory calcitriol cause negative feedback control [8]. The renal CYP27B1 expression is controlled by PTH and FGF23 [1]. Thus, this system is intricately connected.

### 1.2. Biological Differences: Hormonal Form in the Blood vs. Target Tissue Calcitriol Concentration and Gene Regulation

Vitamin D/calcitriol receptors (CTR, also call VDR) and the enzyme CYP27B1 are abundant in peripheral target tissue cells, including immune and vascular cells that hydroxylate 25(OH)D to 1,25(OH)_2_D [9,10]. However, generating sufficient intracellular calcitriol in target tissue cells requires maintaining serum 25(OH)D concentrations above 50 ng/mL. Below which, the functions of the immune system are suboptimal. In a feedback control system, the active metabolites of vitamin D are inactivated by cytochrome enzymes, 24,25-hydroxylase (CYP24A1) and CYP3A4.

Once sufficient calcitriol is generated in these cells, it interacts with CTR and translocates the complex into the nucleus. In the nucleus, the 1,25(OH)_2_D—CTR complex heterodimerises with the retinoid-X receptor (RXR). These complexes bind to vitamin D response elements (VDREs) on target genes and recruit transcription factors, corepressors, and coactivators, which modulate the transcription of DNA. Interactions of the receptor complex with DNA binding domains initiate gene transcription, leading to the regulation of over one thousand genes [11]. However, various tissues have different serum 25(OH)D thresholds for proper function [12]. For example, a serum 25(OH)D concentration of 20 ng/mL is sufficient for renal tubular cell conversion into hormonal calcitriol for musculoskeletal functions [10,12,13,14].

Whereas controlling cancer cell growth, autoimmunity, facilitating autocrine/ intracrine and paracrine signalling, and robust immune responses require a longer-term maintenance of circulatory 25(OH)D concentrations above 50 ng/mL [8,15,16]. In addition, such serum 25(OH)D concentrations are necessary to overcome infections and control several chronic disorders [12]. As described in the next section, 25(OH)D and 1,25(OH)_2_D also mediate rapid non-genomic actions that are vital for some physiological actions, including endocytotic cellular entry of precursors, the integrity of tight junctions in epithelial and endothelial cells, and immune responses [9]. It discusses the mechanisms involved in vitamin Ds’ (calcifediol and calcitriol) rapid, non-transcriptional functions that work through membrane receptors.

### 1.3. Non-Genomic Actions of Vitamin D

Rapid actions of steroid hormones, including calcitriol, occur via membrane-associated CTR and protein disulphide isomerase, a member-3 (Pdia3) receptor family [8]. This system should be considered a part of the vitamin D endocrine system. Such mechanisms are also present with other steroidal hormones [1]. While biological actions from conventional (canonical) genomic actions of steroidal hormones, including calcitriol, take hours to days, manifesting membrane-based non-genomic actions of calcitriol and calcifediol take only minutes.

Moreover, the non-transcriptional actions are not affected by inhibitors of transcription or protein synthesis [2]. These explain the non-involvement of the genomic mechanisms of the rapid action of lipid-soluble steroid hormones [3]. The cubilin-mediated endocytosis of vitamin D is another example of vitamin D membrane–receptor-associated rapid actions of vitamin D. The article by Donate et al., 2022 describes several non-genomic rapid actions of vitamin D [17,18,19].

Endothelial instability and vascular leaks are associated with certain diseases, including infections are prevented by D_3_ supplementation or its active metabolites. Data suggested that the non-genomic actions of D_3_ are a critical component for mediating epithelial and endothelial cell stability, independent of the common canonical vitamin D-related transcription pathways [9]. Vitamin D and its two common metabolites, 25(OH)D and 1,25(OH)_2_D, have similar potency for these rapid actions. Consequently, deficiency of D_3_ weakens this protective epithelial barrier, causing vascular dysfunction, fluid leakage, and the dissemination of infections [9], leading to septicaemia [10]. Thus, D_3_-mediated nongenomic, non-transcriptional mechanisms facilitate suppressing infections and inflammation and prevent destabilisation of the endothelial and epithelial cells. The provision of active metabolites, calcifediol or calcitriol, is insufficient to overcome.

The non-genomic effects of vitamin D are reported in renal tissues obtained from those with kidney failure. In addition to taking standard calcitriol replacement therapy, ingestion of vitamin D_3_ (cholecalciferol) and/or calcifediol) improve the quality of life and survival of persons with CKD due to any cause [4]. Another example of the non-genomic actions of vitamin D.

Vitamin D deficiency [i.e., low vitamin D and 25(OH)D in the circulation] impairs the beneficial clinical outcomes of vitamin D, despite having normal circulatory concentrations of calcitriol. Unlike D_3_ and 25(OH)D, the circulatory calcitriol does not subject to endocytosis or internalisation into peripheral target cells like immune cells and those cells involved in metabolism. Thus, the hormonal form of calcitriol in the blood has no tangible possibility of initiating genome-mediated outcomes in immune cells. Consequently, hypovitaminosis D, as described above, increases the vulnerability and severity of chronic diseases and infections, leading to high incidences of complications and premature deaths [5].

### 1.4. The Importance of Administration of Vitamin D_3_ in Those with Renal Failure

Vitamin D has pleiotropic effects on body systems, especially the immune, cardiovascular, neurological, and renal systems. Patients with chronic kidney disease (CKD) have insufficient vitamin D, 25(OH)D and 1,25(OH)_2_D. This is due to issues such as gastrointestinal malabsorption, increased catabolism, and impaired renal 1α-hydroxylation. The latter is partly due to hyperphosphatemia and elevated fibroblast growth factor-23 (FGF-23) levels, negatively controlling calcitriol production in renal tubular cells [6].

Abnormalities of vitamin D metabolism lead to secondary hyperparathyroidism and bone loss that responds to calcitriol administration [4]. However, administration of parental vitamin D_3_ in conjunction with calcitriol, the quality of life and survival significantly increased in these patients. As described above, increased expression of CYP24A1 increases the clearance of vitamin D and its active metabolites. This increases the ratio of serum “24,25-dihydroxyvitamin D to calcifediol plus calcitriol” (known as vitamin D catabolic ratio). Higher ratios are associated with increased all-cause mortality [7].

### 1.5. The Rationale for the Need for Universal Minimum Serum 25(OH)D Concentration

Based on many basic and clinical research publications, we estimated that approximately 75% of human immune system functions depend on maintaining a healthy, physiological serum 25(OH)D concentration. The cumulative data over the past decade confirm that the required minimum serum 25(OH)D concentration for immune system functions is 50 ng/mL [20,21,22,23]. Multiple data sets have validated the need for such a universal minimum 25(OH)D concentration supporting humans’ overall health and well-being. Based on numerous validated scientific data, the author advocates that 50 ng/mL as the lower limit of the new global standard for “vitamin D sufficiency,” which covers approximately 98% of medical disorders affecting humans.

The multiple organs- or disease-specific minimum serum 25(OH)D concentrations recommended published are confusing [3,12,14]. Therefore, it is helpful to have a universally applicable minimum serum 25(OH)D concentration that can overcome vitamin D deficiency-related diseases. Universal minimum serum 25(OH)D concentration required 50 ng/mL, is safe and efficacious with a therapeutic range between 50 to 80 ng/mL. Maintaining such levels neither requires regular monitoring of serum 25(OH)D concentrations nor leads to adverse effects [24]. It is the best defence for preventing diseases, especially infections, minimising immune dysfunctions like autoimmune disorders, cancers, and reducing complications.

## 2. Vitamin D—Serum 25(OH)D Concentrations Necessary to Overcome Infections

Regular sun exposure can raise circulatory vitamin D_3_ and 25(OH)D concentrations above 30 ng/mL. However, modern lifestyles, clothing, the migration of populations away from sunny climatic conditions, predominantly indoor lifestyles and increasing sun avoidance behaviour prevent achieving it. Aside from certain fatty fish caught in the wild, sun-exposed mushrooms, and fortified foods, vitamin D from dietary sources is negligible. Neither diet nor vitamin D in multi-vitamin tablets can compensate for the lack of adequate daily sun exposure [25]. Therefore, one should not rely on obtaining adequate vitamin D supplements through multivitamin tablets until the proper doses are included. Even with casual sun exposure and taking multivitamin supplements, it is hard to achieve and maintain serum 25(OH)D concentration above 50 ng/mL in the absence of supplements.

### 2.1. Vitamin D Deficiency Weakens Immune Defences against Infection

Inadequate exposure to ultraviolet (UVB) rays is the main reason for the global pandemic of hypovitaminosis D [1]. Avoiding sun exposure and overuse of sunscreens add to the problem [2,8,25,26]. Because of the reasons mentioned above, even regular sun exposure alone is unlikely to generate sufficient circulating D_3_ and 25(OH)D concentrations necessary for robust immune responses. Consequently, many need vitamin D supplements to stay healthy. The prevalence of hypovitaminosis D is highest during the winter. Besides, the two years of intermittent unwarranted lockdowns during the recent COVID pandemic significantly increased poverty and associated malnutrition, worsened the worldwide vitamin D deficiency and increased morbidity and mortality.

The mentioned lockdowns-associated inactivity, alcohol abuse, and obesity contributed to the increased prevalence of vitamin D deficiency. Lockdowns and curfews lowered mean serum 25(OH)D concentration, thus, significantly increasing the invulnerability to the SARS-CoV-2 virus, its complications, and deaths [26,27]. Nutritional status is one of the critical aspects responsible for overall health and well-being, including maintaining proper immune system functioning [17,28]. In addition to vitamin D, other essential micronutrients, such as zinc, magnesium, omega-3 fatty acids, quercetin, etc., are also necessary to optimise the functions of the endocrine system. Zinc and magnesium are also necessary for the proper biological activity of calcitriol, its receptors, and the immune system [27,29,30,31]. Insufficient intake of these worsens clinical outcomes of hypovitaminosis D.

### 2.2. Barriers to the Administration of Repeated/Cyclical High Doses of Vitamin D

Despite the benefits, some studies report that substantial repeated bolus doses of vitamin D fail to achieve goals, such as preventing rickets [10]; and may even increase falls. Suboptimal clinical outcomes may occur secondary to peaks (i.e., marked fluctuations) of serum 25(OH)D concentrations. The mechanisms include the downregulation of renal calcitriol formation and upregulating of 24-hydroxylase enzyme (CYP24A1) [32,33].

The failure to raise serum 25(OH)D could also occur due to gastrointestinal malabsorption or suboptimal hepatic hydroxylation. However, there is no evidence that a single dose of vitamin D_3_ up to 500,000 IU_,_ downregulating CYP27B1, hindering the conversion of 25(OH)D to calcitriol, or enhances the expression of CYP24A1 in immune cells [34,35], or any other undesirable known effects [22,36,37].

However, because of the shorter half-life, when high bolus doses of vitamin D are administered more than three weeks apart (17 days or more), significant fluctuations of concentrations of 25(OH)D and 1,25(OH)_2_D occur in the bloodstream and in target tissue cells. This failure to stabilise and sustain blood and target tissue concentrations (e.g., prostate gland) and over-expression of 24-hydroxylase reduce serum 25(OH)D and calcitriol concentrations, lessening their biological functions. Therefore, the infrequent administration of vitamin D should not be more than 17 days: for practical purposes, it should not be more than every two weeks.

Those mentioned above increase the catabolism of calcitriol and reduce its endocrine production in renal tubular cells [38]. However, it does not necessarily impair the synthesis of calcitriol in peripheral target tissue cells. Nevertheless, the hydroxylation of 25(OH)D to 1,25(OH)_2_D in peripheral target tissues/cells like immune cells will reduce when the interval of vitamin D administration exceeds two weeks, or an insufficient dose of vitamin D is administered. As a rule of thumb, the higher the length between doses, the leaser beneficial biological activity. This volatility of circulating 25(OH)D and 1,25(OH)_2_D concentrations could activate the catabolic pathways of vitamin D metabolism. Consequently, it can impair clinical outcomes, starting with musculoskeletal dysfunctions and extending to other tissues with severe vitamin D deficiency.

It is not the higher serum 25(OH)D concentrations per se but the significant fluctuation of vitamin D metabolites in the bloodstream and in tissues that causes adverse clinical outcomes following repeated bolus doses of vitamin D. Many data sets confirmed that maintaining serum 25(OH)D concentrations above 40 ng mL significantly reduce comorbidities [38]. However, to overcome infections and prevention of complications, cancer prevention, and reduce all-cause mortality, one needs to maintain circulatory 25(OH)D concentrations of more than 50 ng/mL.

### 2.3. Calcitriol or Its Analogues Should Not Be Used as Vitamin D Supplements

While the circulatory half-life of calcifediol is between two to three weeks [39], calcitriol lasts only a few hours [40]. Excess or peaks of calcitriol in the blood shut down the expression and secretion of PTH and increases the feedback expression of the 24-hydroxylase (CYP24A1) enzyme that catabolises calcifediol and calcitriol into their inactive 24-hydroxylated metabolic products [41,42]. Similarly, this feedback mechanism is activated by repeated high (bolus) doses of vitamin D; thus, should be avoided.

Unlike vitamin D_3_ and 25(OH)D, the therapeutic window (i.e., ED 50) for calcitriol is narrow. The dose of calcitriol required for 50% of the population to attain 50% of the desired response (i.e., be 50% effective) is close to its toxic dose. Consequently, calcitriol administration has a substantial risk of adverse effects, such as hypercalcaemia and hypercalciuria, downregulating CYP2R1 through increased FGF23 production and the upregulation of tissue vitamin D-24-hydroxylase catabolic enzyme.

Since exogenous calcitriol does not enter immune cells [43], there is no rationale for administering calcitriol at any dose to boost the immune system or as an adjunct therapy for infections [10]. Administering calcitriol does not benefit in controlling infections; it increases the risk of significant adverse effects. Exceptions are those with hypoparathyroidism and advanced renal failure. Therefore, it is unsurprising to see clinical outcomes failures using calcitriol in several recently published randomised controlled clinical studies (RCTs) on subjects with sepsis and COVID-19. Outcome failures were predictable due to using the wrong medication (no better than a placebo) and faulty study design [41,42].

**Figure 1:**
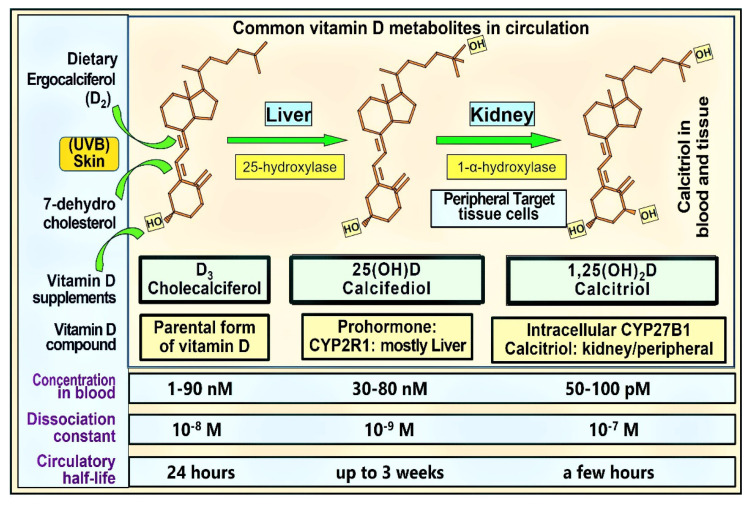
The figure illustrates the structures of the three most familiar, clinically relevant vitamin D analogues, their primary in situ sites of generation, respective hydroxylating enzymes and reported concentrations in the bloodstream. While in humans, the circulatory levels of D_3_ and 25(OH)D_3_ are present in the nanomolar range, 1,25(OH)_2_D is in the picomolar range: approximately a thousand-fold lesser concentration (numerical data from Hollis et al., added in the last two rows, [9]).

With exorbitant cost, lack of immune cell uptake, and negative feedback down-regulatory mechanisms, neither calcitriol nor its 1α-analogues should be used as vitamin D supplements or treatment for infections, including COVID-19. Figure 1 illustrates the structure–functions of the commonly used vitamin D analogues: circulatory levels, dissociation constants, and circulatory half-lives.

Calcitriol has the highest percentage of free (unbound or loosely bound to VDBP) components in the circulation out of the three commonest vitamin D metabolites. However, since the concentration of calcitriol is about a thousand times less than D_3_ and 25(OH)D, its absolute free quantity is very low and insufficient to enter immune cells. While present in similar concentrations in the circulation, 25(OH)D is more tightly bound to VDBP than vitamin D_3_. Therefore, quantitatively and percentage-wise, when taken daily, due to its relatively lower affinity to VDBP, more D_3_ is available to diffuse/endocytose into target cells compared to 25(OH)D [9]. This highlights the importance of the availability of D_3_ as a precursor to generating calcitriol in peripheral target cells. However, this benefit is lost if inappropriately relying on 25(OH)D or 1,25(OH)_2_D and ignoring the intake of parental D_3_. Consequently, despite its short-term advantages, calcifediol should not be routinely used as a vitamin D supplement.

The diffusion coefficients (including endocytosis) of these three compounds transfer into target cells and correlate with their circulating concentrations, half-lives, and affinity to VDBP. Based on these, one could imagine that approximately equal amounts of D_3_ and 25(OH)D enter into peripheral target cells. However, these two components can compensate for each other.

In addition, as described above, renal tubular-, musculoskeletal-, and fat cells have an in-built active transport mechanism for the cellular entry of D_3_ and 25(OH)D—the megalin–cubilin endocytotic receptor system [11]. This endocytotic system is vital in delivering vitamin D and 25(OH)D not only to their central target, proximal renal tubular cells in the kidney, but also into Chief cells in the parathyroid glands, for its endocrine functions, through 1α-hydroxylation [11]. These molecules use a similar mechanism to enter muscle and fat cells [12].

### 2.4. Benefits of Raising Population Vitamin D Sufficiency during a Pandemic 

An inverse relationship was reported between pre-pandemic serum 25(OH)D concentration and a 50% reduction of risks of COVID-19 in 190,000 adult Americans across fifty states [44]. In this group of adults, the risk of infection and complications are significantly and inversely related to pre-pandemic vitamin D status [15,45]. Others have reported the relationships between the serum 25(OH)D concentration and infection risk reductions.

Long-term vitamin D repletion via supplementation is the most cost-effective and practical approach for maintaining a robust immunity in the population, reducing disease burdens and healthcare costs [20,46]. The higher the population immunity achieved through maintaining a higher serum 25(OH)D concentration, the lower the risks of symptomatic infections and complications from epidemics and pandemics like COVID-19, even in the absence of vaccination. This is one of the explanations for lower complications, hospitalisation and death rates reported in fewer COVID-vaccinated countries.

**Figure 2:**
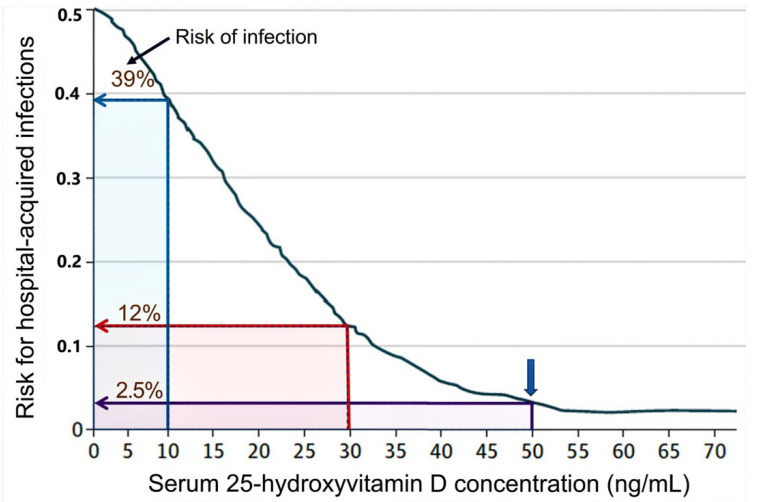
An inverse relationship between vitamin D status and the risk of hospital-acquired infections. The maximum reduction of the infection rate was achieved when the serum 25(OH)D concentration exceeded 50 ng/mL (i.e., the background rate). This supports that 50 ng/mL as the minimum level required to overcome infections. Data presented as a multivariable logistic regression analysis with a locally weighted scatterplot. To demonstrate differences in hospitalisation risks, the percentage risk of infections at serum 25(OH)D concentrations of 10, 30, and 50 ng/mL are illustrated. The blue arrow shows a background risk of approximately 2.5% (modified after Quraishi, S.A, et al., 2014) [15].

An example of a strong association between serum 25(OH)D concentration and infections is illustrated in Figure 2. This prospective clinical study assessed the relationship between serum 25(OH)D concentration and the risk of hospital-acquired infection rates. This clinical study also highlighted the importance of maintaining serum 25(OH)D concentrations over 50 ng/mL for reducing infections to the background rate [15,20].

## 3. Vitamin D and Immune Functions

Low serum 25(OH)D concentration at hospital admission predicts the severity and deaths from COVID-19 [44,45,47,48,49,50]. Low pre-pandemic vitamin D status is dose-dependently and inversely associated with higher risks of contracting COVID-19 and its severity [44,45]. The importance of vitamin D adequacy on adaptive immunity is illustrated by serum 25(OH) D-dependent increased antibody formation following COVID immunisation [47,48].

### 3.1. Role of Vitamin D in Immune Protection against Infections

There was an opportunity to strategically use the combination immune protection derived from the broader and longer-lasting natural immunity after infection with the post-vaccination derived immunity (narrow and short-lasting) to curtail the spread, complications, and death from SARS-CoV-2 and to eradicate the virus. However, the global authorities disregarded such a practical approach. Instead, because of the ongoing global political trends, the WHO- and CDC opted to recommend and entirely depend on COVID vaccines, disregarding the value of natural immunity. That prevented achieving herd immunity and the eradication of SARS-CoV-2.

Emerging global data demonstrate a continual loss of efficacy, despite increasing numbers of booster doses of COVID-19 vaccines. The loss of effectiveness of COVID vaccines is partly due to the development of immune tolerance and increased mutations, especially in the Spike protein region of the SARS-CoV-2 virus, which led to immune evasion [51,52]. This is also associated with increased complication rates due to antigen-enhancing reactions among vaccinated [53] (https://openvaers.com/covid-data (accessed on 4 May 2022)). While mRNA-based COVID vaccines reduce the severity and complications, the specificity of neutralization antibodies is narrow and directed only against portions of Spike protein and does not produce mucosal immunity. Consequently, such vaccines do not prevent the spread of SARS-CoV-2 or re-infection.

**Figure 3:**
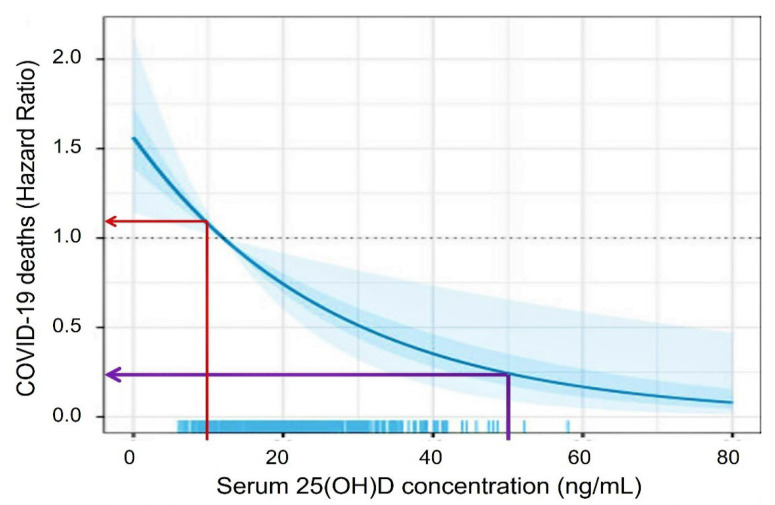
Post-estimation simulation of 25(OH)D concentrations using 15 and 50 ng/mL as the cut-offs predict more than four-fold higher mortality from COVID-19. Data adjusted for age, sex, BMI, C-reactive protein, D-dimer, oxygen saturation, and chronic diseases, such as type 2 diabetes and chronic kidney disease (modified after Vanegas-Cedillo, P. et al., 2022) [55].

Vitamin D sufficiency improves immune functions [54]. It significantly reduces the risk of viral and bacterial respiratory infections [55,56,57] and lowers the risk of adverse outcomes from COVID-19: including mortality from SARS-CoV-2 [55] and complications following immunisation. As illustrated in Figure 3, the death rate from COVID-19 is four-fold less, having a serum 25(OH)D concentration above 50 ng/mL [55]. A single bolus dose of vitamin D or calcifediol discussed above or an upfront loading dose of vitamin D discussed later can rapidly reach the mentioned therapeutic serum 25(OH)D concentration and, thus, improve clinical outcomes. The methods mentioned in Section 5 below for rapidly achieving higher serum 25(OH)D concentrations are practical and effective in rectifying severe vitamin D deficiency and boosting the immune system. They are helpful in emergencies to achieve rapidly raise 25(OH)D concentration, as in COVID-19 [15,20,58,59,60].

### 3.2. Bioavailable D_3_ and 25(OH)D for Intracellular Synthesis of Calcitriol in Target Cells

A sustained serum 25(OH)D concentration above 50 ng/mL [20] is crucial for maintaining robust innate and adaptive responses preventing immune dysregulation and hyperinflammatory responses. In part, clinical validation of this is illustrated in Figure 2 and Figure 3 [20,61,62,63]. Because of the technical and practical difficulties in measuring intracellular vitamin D metabolites (e.g., in immune cells) under physiological conditions, the biological sensitivity or the cut-off levels is unknown.

VDBP and other binding proteins are synthesised in the liver and regulated by glucocorticoids, oestrogen, and inflammatory cytokines. Of the circulating 25(OH)D, bioavailable 25(OH)D (free plus that bound to VDBP) accounts for approximately 15% of the total. This portion is available to enter peripheral target cells via a concentration gradient and endocytosis [41,64]. The bioavailable component of vitamin D_3_ has a lower affinity to VDBP than 25(OH)D. Therefore, when the circulating concentrations of D_3_ and 25(OH)D are similar, a higher quantity of D_3_ can enter immune cells. As mentioned, in kidneys, muscle, and fatty tissues, VDBP-bound vitamin D and 25(OH)D enters via the active process, a megalin/cubilin transport complex through endocytosis [65]. This is another example of a vitamin D-membrane receptor mechanism. This active endocytosis process is an essential prerequisite for synthesising the hormonal form of calcitriol, 1,25(OH)_2_D [64].

Direct measurements of intracellular concentrations of vitamin D metabolites are neither dependable nor feasible. The concentration of the hormonal form of calcitriol in the circulation is approximately 0.045 ng/mL. However, the concentration of calcitriol necessary for intracrine/autocrine and paracrine signalling in immune cells is estimated at approximately 1.0 ng/mL. This is more than two orders of magnitude higher than the circulatory calcitriol concentration. Therefore, circulatory calcitriol is unlikely to enter immune cells against such a high gradient.

### 3.3. Autocrine and Paracrine Signalling in Immune Cells

Unlike the renal tubular cell entry of calcitriol, the entry of vitamin D and 25(OH)D to peripheral target cells, such as immune cells, for their autocrine/intracrine functions requires a higher circulating concentration of these compounds. Therefore, peripheral target cells require a higher gradient of D_3_ and serum 25(OH)D concentration (i.e., above 50 ng/mL) to enter immune cells than renal tubular cells. The failure to maintain circulating vitamin D levels due to periodic inadequacy of D_3_ and 25(OH)D concentrations (i.e., the trough portions) secondary to intermittent, repeated administration of vitamin D bolus doses beyond two weeks intervals (or for other reasons for the fluctuation of circulatory levels) has proven ineffective for intended beneficial effects from vitamin D, as shown in some recent clinical trials [13].

The protocols described in Table 1 and Table 2 are for one-time bolus or loading doses to boost the serum levels and the immune system; these are not intended to administer repeatedly [15]. Therefore, it does not subject to the mentioned troughs in circulatory levels and, hence avoids the adverse effects discussed above. Considering the shorter half-life of vitamin D in the circulation, administration of daily or weekly doses is preferable. Nevertheless, the adherence (compliance) to once-a-week therapy is significantly better than daily or once-in-two-week intakes because it is easy to remember, as with other infrequently taken medication treatments, as with once-a-week bisphosphonate therapy for osteoporosis compared to daily intake. However, when initiating a regimen, the patient’s preference and travel patterns, should be considered for better adherence to therapy. During the designing of study protocols of RCTs, the expected compliance should be considered that generates beneficial clinical outcomes.

**Figure 4:**
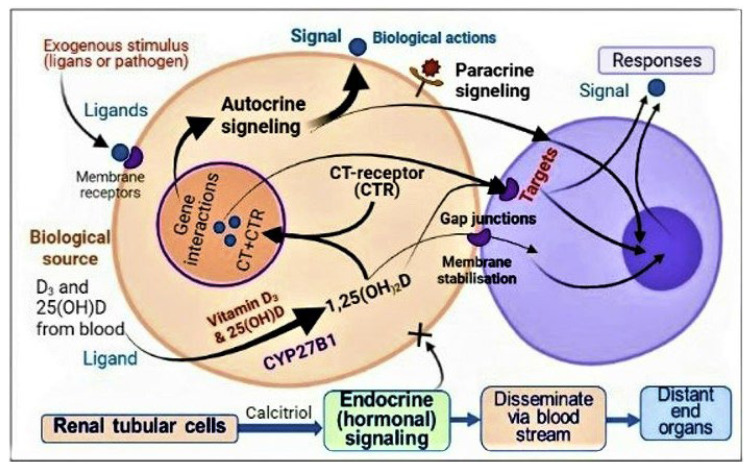
Illustrates the intracellular generation of calcitriol in immune cells that activate autocrine and paracrine signalling mechanisms. Once vitamin D and 25(OH)D enter the peripheral target cells (e.g., immune cells) through passive diffusion and/or active endocytosis, CYP2R1 and CYP27B1 hydroxylate them to form 1,25(OH)_2_D (calcitriol). Immune cells contain abundant CYP2R1, CYP27B1, and calcitriol receptors (CTR). Calcitriol-CTR complexes enter the nucleus and interact with the genome, regulating up or down the expression of genes as described in Section 1.2. This leads to an increased expression of anti-microbial peptides, antibodies, etc., and decreases the expression of inflammatory and oxidant cytokines.

In addition, most people worldwide have serum 25(OH)D concentrations between 15 and 30 ng/mL (i.e., vitamin D deficiency status), which prevents sufficient D_3_ and 25(OH)D from entering immune cells [66,67]. Therefore, it is not surprising that they have weak immune systems. Therefore, in acute situations like sepsis and COVID-19, it is necessary to raise both serum vitamin D_3_ and 25(OH)D to sufficient concentrations quickly, enabling these molecules to enter immune and other target cells to generate intracellular calcitriol [62,63,68] (Figure 4).

### 3.4. Improve Vitamin D Status, Reduce the Risk of COVID-19 Complications 

Vitamin D sufficiency heightens the activity of both the innate and adaptive immune systems [56,57]. Several clinical studies reported the significant benefits of vitamin D supplementation in acute respiratory tract infections [24,69,70,71]. Multiple RCTs reported that vitamin D supplementation strengthens the immune responses against seasonal/winter-associated respiratory viral infections with a 50% reduction in incidence and severity [57,72,73].

**Figure 5:**
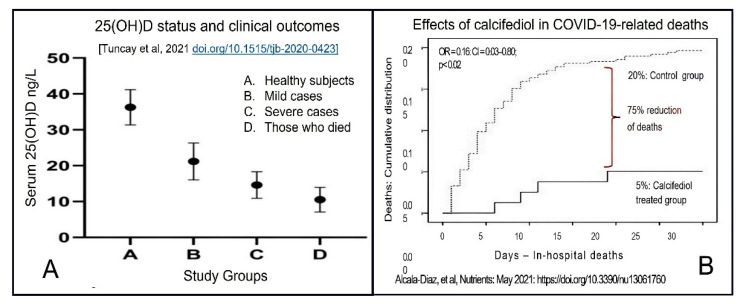
(**A**). A significant inverse correlation between serum 25(OH)D concentrations and the severity and mortality from COVID-19 (after Tuncay et al., 2021) [80]. (**B**). The cumulative in-hospital mortality of those treated with calcifediol versus a control group. Mortality was 5% in the treated group versus 20% in the placebo group: a reduction of 75% in mortality rate in the calcifediol-treated group (After Alcala-Diaz, et al., 2021) [81].

Many studies have reported a strong inverse association between serum 25(OH)D concentration and COVID-19 severity and mortality [74,75,76,77]. Partially activated vitamin D, 25(OH)D (calcifediol) administered orally, acts within four hours of administration and is convenient to use. Doses between 0.5 and 1.0 mg can raise the serum 25(OH)D to therapeutic concentration (above 50 ng/mL) within four hours instead of a few days as with regular, parental vitamin D_3_ [78,79]. Such doses of calcifediol, however, do not trigger downregulation of calcitriol production by autoregulatory mechanisms [10]. Figure 5 illustrates the superior efficacy (i.e., better clinical outcomes—reduction of deaths) of data from an RCT, using 0.523 mg of calcifediol in those with moderate COVID-19. The study demonstrated that calcifediol lowered mortality by 75% compared with the control group [79,80].

**Figure 6:**
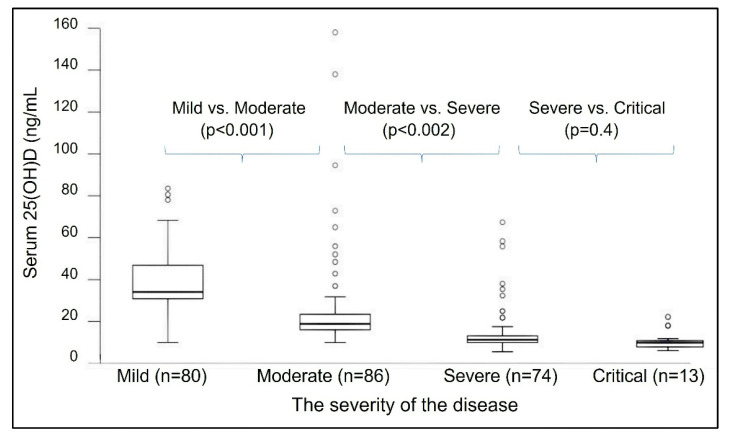
Box plots [range of serum 25(OH)D concentrations of 50% of the cases, within the interquartile range] indicated a significant association between the severity of COVID-19 and pre-infection serum 25(OH)D concentration (*n* = 253). The comparisons were made between mean vitamin 25(OH)D concentrations with four severity categories of COVID-19—mild to critically ill, using the WHO definition of severity (WHO/2019-nCoV/clinical/2020.5). The *p*-values are presented after multiple-category comparisons using the nonparametric Kruskal–Wallis test. A Mann–Whitney U-test compared 25(OH)D concentrations with the mean rank of neighbouring sequential categories, and individual outliers are presented with open circles (after Drorl et al., 2022) [45].

Other studies report that the minimum serum of 25(OH)D concentration needed for better health is not 30 ng/mL but 40 ng/mL [71,82,83]. However, for optimal immune responses, cancer prevention, and reduced all-cause mortality, the minimum serum 25(OH)D concentration necessary is 50 ng/mL [84] (Figure 2 and Figure 3). Figure 6 illustrates the inverse relationship between COVID-19-related disease severity and serum 25(OH)D concentrations prior to infection [45].

## 4. Summary of Evidence from Clinical Trails

Many observational and some RCTs have demonstrated strong inverse correlations between serum 25(OH)D concentrations and COVID-19 risks [44,45,84]: incidence, severity, rates of ICU admissions, and mortality [45,47,56,57,84,85,86]. Vitamin D supplementation alone and in combination with calcifediol significantly reduced complications [35,45,47,87], hospitalisations, and mortality [85,86]. It is noteworthy that, unlike vitamin D_3_, calcifediol needs a prescription, and physicians are not yet familiar with its benefits and how to use it.

**Figure 7:**
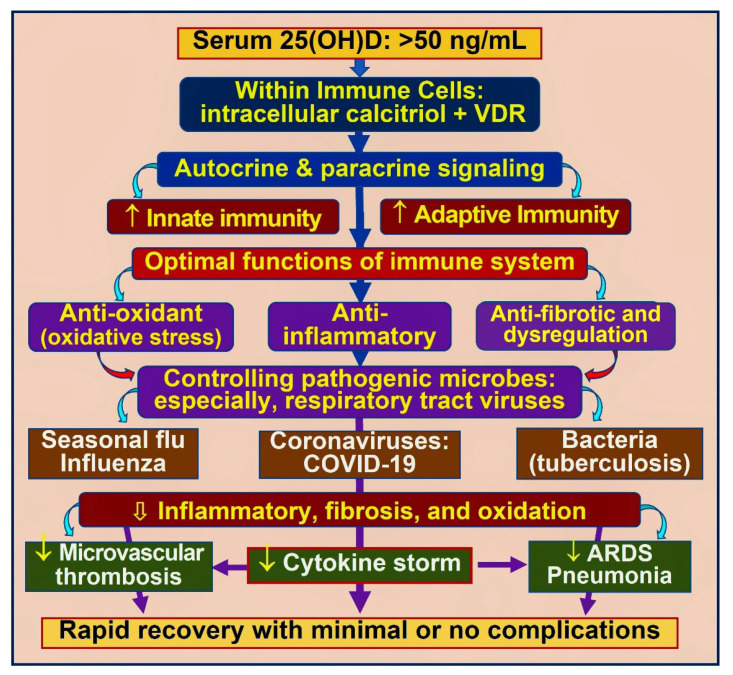
The upregulation of the innate and adaptive immune functions of immune cells by calcitriol. Examples include anti-inflammatory and antioxidant benefits, downregulation of viral replication, reduced risk of cytokine storms, microvascular thrombosis, and acute respiratory distress syndrome (ARDS).

Most people who developed post-COVID syndrome had hypovitaminosis at the time of getting infected with SARS-CoV-2 or hospitalisation. Figure 7 illustrates the complex interactions of vitamin D in modulating the innate and adaptive immune systems. Vitamin D is also neuroprotective and minimises the occurrence and severity of longer-term complications, such as the post-COVID syndrome [19,88]. Many RCTs that compared calcifediol versus controls demonstrated a significant reduction in COVID-19 complications, hospitalisations, and mortality [45,81,86,87]: the RCT by Alcala Diax et al., used once-a-week calcifediol [81] (Figure 5). Despite these positive clinical studies, unsurprisingly, poorly designed RCTs reported outcome failures [89,90].

Figure 7 also illustrates the importance of maintaining serum 25(OH)D concentration above 50 ng/mL for immune cells to function appropriately and mediate their crucial autocrine and paracrine functions. Optimal functioning or innate and adaptive immune responses would prevent cytokine storms and complications from infections, leading to a rapid recovery from the illness. The following section discusses the rare potential complications from excess vitamin D.

### 4.1. Potential Adverse Effects of Vitamin D through Hypercalcaemia

In the community, hypercalcemia is most commonly due to (mostly) undiagnosed hyperparathyroidism. However, in hospital settings, it is commonly related to cancer due to the inappropriate secretion of parathyroid hormone-related peptide (PTHrP) and metastatic bone disease [91,92]. Other causes of hypercalcemia, such as genetic mutations of CYP24A1, etc., are extremely rare [93,94].

When hypercalcaemia is detected, calcium and vitamin D administration should stop, and the root cause should be investigated. The suggested vitamin D doses in Table 1 and Table 2 below are safe unless initial (the baseline) serum 25(OH)D concentrations exceed 60 ng/mL (this is a very smaller proportion of the public), currently taking high doses such as 50,000 IU more frequently than once in two weeks, or have sporadic genetic disorders [45,86,95,96,97,98]. Those categories of people are likely to have higher serum 25(OH)D concentrations or may have higher sensitivity; thus, they do not need vitamin D or calcium supplementation.

However, one should be cautious when serum 25(OH)D levels exceed 150 ng/mL [99]. A few studies reported adverse effects from increased circulatory ionised calcium due to enhanced gastrointestinal and renal absorption following extremely high serum 25(OH)D (i.e., over 200 ng/mL), which is associated with high serum 1,25(OH)_2_D concentrations. This can be avoided by stopping calcium supplements and significantly reducing dietary calcium intake, as described in the Coimbra Protocol [37].

### 4.2. Therapeutic Interventions—Recommendation for High-Dose Vitamin D

Higher-dose vitamin D_3_ capsules (generally, 50,000 IU; 60,000 IU capsules in India) are available in most countries at a nominal cost. Few studies have reported that consuming daily vitamin D_3_ doses of 20,000 IU [100,101] to 50,000 IU [98] is devoid of adverse effects, like hypercalcemia or hypercalciuria, even in children [36]. However, such is not recommended for the general public unless a specialist with expertise prescribed such for a specific reason like psoriasis or migraine headaches. People who consume such high daily doses must work under the direct supervision of an experienced physician with regular blood checks.

Nevertheless, higher doses of oral vitamin D are indicated in specific clinical conditions, such as infections [45,86,95,96,97,98], including COVID-19 [86] (see Section 5), which provides overwhelming benefits. Other indications for prescribing high doses of vitamin D are related to specific medical issues managed by relevant experts. Examples are vitamin D-resistant disorders (e.g., vitamin D-resistant rickets; genetic abnormalities of CYP enzymes), hypoparathyroidism, genetic and acquired hypophosphataemic osteomalacia, renal osteodystrophy, migraine, psoriasis, malabsorption, obesity, osteomalacia secondary to hepatic impairment, etc.

### 4.3. Cost-Effective Strategies to Maintain a Robust Immune System via Vitamin D

The essential public health measures for preventing viral respiratory infections are wearing N95 or KN95 type facemasks, avoiding closed indoor spaces and crowd gatherings and keeping safe distances between people. The goal is to prevent the entry of large quantities of viruses into human cells and their replications and minimise viremia. Steps to blocking the occurrence of symptomatic disease are prevention or avoidance of viral entry through cell membrane-bound ACE-2 receptors (entering mostly via the respiratory tract), inhibiting intracellular viral replication, in vivo viral neutralisation and destruction, and enhanced elimination of viruses from the body through a robust immune system.

The above is achievable via the combination of (A) natural boosting of the immune system by raising the individual’s and population’s serum 25(OH)D to prevent immune dysfunction and overcome infections, (B) maintaining a robust immune system in the population with sun exposure and vitamin D sufficiency, (C) protecting the vulnerable—the elderly and those with comorbidities, (D) value and utilise the natural immunity of those who recovered from COVID-19, and (E) billions of people immunised with vaccines. Health authorities could have collectively leveraged the above into a practical path to attain herd immunity, geared toward eradicating the SARS-CoV-2 virus. However, they rejected such approaches and thus, this failed to materialize.

Calcifediol with a second hydroxylation is more water-soluble than D_3_. Therefore, it gets absorbed more rapidly, even without lipid-based intestinal absorption mechanisms [102]. Consequently, even in the presence of gastrointestinal malabsorptive issues (e.g., after bariatric surgery) [103], abnormal liver function, and ongoing severe illness, oral calcifediol rapidly increases serum 25(OH)D concentrations, enabling its entry into target cells.

All peripheral target cells, including immune and vascular cells expressing high CTR concentrations and CYP2R1, thus, can convert 25(OH)D into calcitriol. In immune cells, this intracellularly generated calcitriol (not the hormonal form in the circulation) combines with its cytosolic CTR receptors to form calcitriol–CTR complexes and translocates into the nucleus to interact with DNA as described above. It initiates autocrine and paracrine signalling in immune cells, as represented in Figure 4.

### 4.4. Mechanisms through Which Vitamin D Control Inflammation

Vitamin D deficiency promotes a pro-inflammatory milieu of Th1 and Th17 cells [16]. In contrast, adequate intracellular calcitriol switches the pro-inflammatory Th1 and Th17 cells into anti-inflammatory Th2 and Treg response cells. The resultant reduced inflammatory cytokine release and increased anti-inflammatory cytokines expression prevent cytokine storm and ARDS [104,105]. The primary reason the Th1 and Th17 lymphocytes remain pro-inflammatory during infections is insufficient D_3_ and 25(OH)D in the circulation.

**Figure 8:**
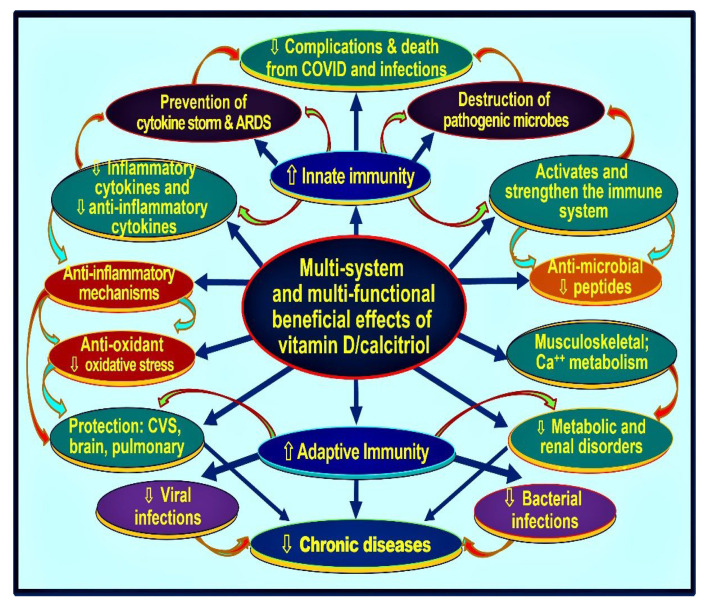
Crucial biological and physiological functions of calcitriol [1,25(OH)2D] in various body systems, focusing on the immune system. Intracellularly generated calcitriol stimulates both innate and adaptive immune systems. Essential immunological functions of vitamin D, such as anti-inflammatory, anti-microbial, and antioxidant activities, enables overcoming invading microbes like bacteria and viruses (depicted in purple ovals). Vitamin D sufficiency positively modulates immune cells (depicted in red ovals), reduces multiplication, increases the destruction of pathogens like SARS-CoV-2, and tightens gap junctions, thus preventing fluid extravasation and the spread of microbes. Not included are, increased expression of ACE-2 and reduced concentration of angiotensin-II, which diminishes the risk of cytokine storm, acute respiratory distress syndrome (ARDS), and death (modified from Wimalawansa, S.J., 2020, [16]).

The calcitriol-driven shut-down program converts Th1 cells into Th2 and Th17 to Treg cells. It redirects pro-inflammatory to anti-inflammatory milieu [16,106]. This permits the immune cells’ intended autocrine and paracrine signalling mechanisms, reducing inflammation and enhancing the expression of anti0inflammatory cytokines, IFN-ƴ, IL-10, etc. [23,107]. Figure 8 is a schematic illustration of the overall effects of vitamin D on various body systems, including modulation of innate and adaptive immune systems.

In addition to its non-genomic antiviral effects [108], calcitriol enhances the transcription of several anti-microbial peptides, such as cathelicidin and defensin. It also stimulates the chemotaxis of immune cells [109] and reduces the severity of COVID-19 [110]. However, individual variations in responses are expected to occur through epigenetic variances [106,111]. Vitamin D also improves the functions of Thαβ CD4+ T lymphocytes, suppresses T17 helper lymphocytes, and increases the expression of IL-10 and virus-specific IgG1 antibodies by activating T-cell-dependent B lymphocytes [112].

### 4.5. How Obesity Causes Hypovitaminosis D, Requiring Higher Doses of Vitamin D?

The triad of obesity, diabetes mellitus, and metabolic syndrome are chronic, low-grade inflammatory conditions [113,114,115]. These conditions are associated with excess intra-abdominal inflammatory fat hypovitaminosis D, and chronic inflammation, which increases complications and premature deaths from myocardial infarction and strokes. Insufficient amounts of bioavailable substrates [circulatory D_3_ and 25(OH)D] entering target immune cells lead to failure to generate intracellular calcitriol. It causes hypovitaminosis-associated generalised hyper-inflammation and oxidation and cytokine storms [116]. Conversely, vitamin D supplements reduce chronic inflammation, control blood sugar, and reduce complications and deaths from these disorders [117,118].

In obesity, intra-abdominal fat cells are in inflammatory status. They produce ‘toxic’ cytokines that suppress CYP2RI in the liver [119]. It partially works via the peroxisome proliferator-activated receptor coactivator 1-a(PGC-1a)/estrogen-related receptor and the glucocorticoid receptor [114]. Besides, fat-cell mediated sequestration, inactivation of D_3_ and calcifediol by CYP24A1 and downregulation of CYP2RI in the liver (and perhaps in peripheral tissues) further reduce circulating 25(OH)D concentration [114,120]. The above explains why persons with obesity are invariably having low serum 25(OH)D concentrations. They require two to four times higher doses of vitamin D on a body weight basis to maintain serum 25(OH)D concentration [9] (describes in Section 5 below).

## 5. Doses of Vitamin D Necessary to Boost Serum 25(OH)D 

Higher than the generally recommended doses of vitamin D are necessary to maintain a robust immune system and reduce the risks of infection, severe complications, deaths, and all-cause mortality [121]. For a 70 kg healthy adult, the recommended vitamin D supplementation to maintain serum 25(OH)D above 50 ng/mL is approximately 5000 IU/day (between 4000 and 7000 IU/day), or 50,000 IU weekly: for persons with lower body weight (low body fat/BMI), 50,000 IU, once in two weeks [4,12,122].

However, the elderly and overweight or obese individuals need two- to four-fold higher amounts of vitamin D to achieve and maintain the above-mentioned target circulatory concentrations, as described below [5]. Those at higher risk of developing infections and have a higher prevalence of hypovitaminosis D, such as obese individuals, the elderly, immune-compromised, and those with comorbidities [123,124], require much higher doses of vitamin D than the average weight adult or a younger person [5,12,122,125,126] (see Section 4.5 above, for explanation).

There is a trend to propagate the use of free 25(OH)D as a better clinical tool than the accepted and validated total 25(OH)D routinely measured in laboratories worldwide. Nevertheless, other reported studies showed no advantage of doing so [127,128]. Besides, there are neither meaningful genetic or racial differences in VDBP concentrations nor the affinity of DBP for the three vitamin D ligands mentioned above.

Consequently, calculated or measured free 25(OH)D equally correlates well with the total 25(OH)D [129]. Also, free 25(OH)D and calcitriol concentrations increase with various diseases such as cirrhosis, making it impossible to interpret free concentrations in a meaningful way [130]. Most importantly, since both free and VDBP-bound forms of D_3_ and 25(OH)D enter target cells like immune cells, they are biologically meaningful. Therefore, the reliance on measured free 25(OH)D components in the circulation does not add value and is misleading in clinical practice; thus, it should be avoided.

### 5.1. Vitamin D Intakes Needed to Maintain Serum 25(OH)D Concentrations > 50 ng/mL

The circulatory half-life of 25(OH)D is less than 20 days (varies from 12 to 24 days, depending on the age and the vitamin D status). Therefore, to maintain desirable, stable therapeutic serum 25(OH)D concentration, vitamin D supplementation should be taken in intervals of no more than two weeks apart [118]. Because the hepatic 25-hydroxylation (CYP2R1) is a rate-limiting step, upfront loading doses, even exceeding 300,000 IU bolus dose, take three to four days to raise serum 25(OH)D concentrations.

However, a single administration dose-dependently maintains a higher serum 25(OH)D concentration for up to three months [2,66,123,124]. For more extended maintenance, daily or weekly maintenance doses are necessary. While a single bolus or a high-loading dose is safe, repeated higher doses upregulate CYP24A1. Therefore, likely to enhance the inactivation (catabolism) of D_3_, 25(OH)D, and 1,25(OH)_2_D. Subsequently, it impairs certain physiological functions of vitamin D, mainly the hormonal functions of vitamin D (see Section 1.1): the latter adverse effects could last up to three months [66].

Therefore, regardless of the loading doses of vitamin D_3_ used to raise serum 25(OH)D, the severely ill, as in those critically ill in the ICU, it may take up to a week to raise their serum 25(OH)D concentration. Therefore, physicians should not rely on parental vitamin D supplements to promptly boost the immune system in severely ill patients [89,131]. It has no benefit but could cause serious adverse effects. In these situations, the administration of oral calcifediol [25(OH)D] between 0.5 and 1.0 mg (0.014 mg/kg body weight) is the most effective and safe way to raise serum 25(OH)D concentrations rapidly and boost the immune system. A straightforward way to calculate the required dose is illustrated in Tabel 1, when the serum 25(OH)D concentration is available.

### 5.2. Practical Ways to Use Higher Doses of Vitamin D_3_ Supplementation

Because of their wide availability, practicality, affordability, and better gastrointestinal absorption, it is recommended to use 50,000 IU D_3_ capsules when higher quantities of vitamin D are needed as replacement doses, which is the most cost-effective approach. A practical way of using these to enhance serum 25(OH)D concentration and maintain it in non-urgent situations, among outpatients, and in the community is illustrated in Table 1. Required numbers of capsules can be taken as a bolus dose of 100,000 to 400,000 IU (single upfront doses) or in divided daily doses with a meal, as illustrated in Table 1.

**Table 1:** When serum vitamin D levels are available, the doses provided in this table can be used for the longer-term maintenance of serum 25(OH)D concentration above 50 ng/mL (125 nmol/L). The table provides the initial bolus dose, weekly dose, frequency, and the duration of administration of oral vitamin D in non-emergency situations, in a non-obese, 70 kg adult. * (modified from Wimalawansa, S.J., 2012) [132,133]).

Serum Vitamin D (ng/mL) **	Vitamin D Dose: Using 50,000 IU Capsules: Initial and Weekly ^$^		Duration (Number of Weeks)	Total Amount Needed to Correct Vit. D, Deficiency (IU, in Millions) ^#^
	Initial Bolus Dose (IU)	Follow-Up: ^$$^ The Number of 50,000 IU Caps/Week		
<10	300,000	×3	8 to 10	1.5 to 1.8
11–15	200,000	×2	8 to 10	1.0 to 1.2
16–20	200,000	×2	6 to 8	0.8 to 1.0
21–30	100,000	× 2	4 to 6	0.5 to 0.7
31–40	100,000	×2	2 to 4	0.3 to 0.5
41–50	100,000	×1	2 to 4	0.2 to 0.3

* A suitable daily or weekly maintenance dose to be started after completing the loading-dose schedule. The dose should be adjusted for those who are overweight (higher) or underweight (lower). ** To convert ng/mL to nmol/L, multiply the amount in ng by 2.5; One µg = 40 IU. ^$^ Mentioned replacement doses can be taken as single, cumulative doses, two to three times a week spread out over a few weeks. ^$$^ From the day one of week two onwards. ^#^ Estimated total vitamin D dose needed to replenish the body stores (i.e., the deficit) is provided in the last column.

Table 1 presents a safe and practical schedule when the serum 25(OH)D concentration is known [132,133]. Since liver 25-hydroxylase CYP enzyme is a rate-limiting factor (but not necessarily in peripheral target cells like immune cells). Therefore, in non-urgent situations, taking D_3_ 50,000 IU capsules spread over a few days provides better absorption and bioavailability than taking the total amount as a bolus dose. As illustrated in Table 1, tissue deficits can be conveniently replenished with 50,000 IU capsules, allowing the body to build up stable serum 25(OH)D concentrations within three to five days and maintain it longer.

In several RCTs, vitamin D has also been administered every two weeks with successful clinical outcomes [28,134,135,136,137,138,139]. However, to efficiently control infections or any other disorder, as described above, it is recommended to administer vitamin D daily or once a week; however, not to increase the frequency of administration for more than ten days.

### 5.3. The Recommended Doses to Maintain Therapeutic Serum 25(OH)D Concentration

For proper functioning of peripheral target cells and to overcome infections, evidence suggests that it is necessary to maintain circulatory 25(OH)D concentrations above 50 ng/mL. This can be achieved by administering vitamin D doses, such as 5000 IU (125 µg/day) or 50,000 IU (1.25 mg) once or once two weeks.

This is in contrast to the daily doses of 400 to 1000 IU (the equivalent of 10 to 25 µg), currently recommended by most governments, Institute of Medicine (IoM)-America [now National Academy of Medicine], the European Food Safety Authority (EFSA), the UK Scientific Advisory Committee for Nutrition (SACN, UK), National Institute of Health & Care Excellence (NICE, UK), and certain European countries, and their recommendations to maintain the minimum circulatory 25(OH)D concentration above 20 ng/mL (50 nmol/L) [20,140].

While laboratory facilities to measure serum 25(OH)D concentrations available in most industrialised countries, they are expensive. In most other countries, measurements of 25(OH)D are not available; thus, relying on shipping samples abroad add to the cost. However, as illustrated in Table 2, the results of serum 25(OH)D concentrations are not essential to assess the needed replacement doses safely. The replacement therapy can be performed without adverse effects when a person has not been on high-dose vitamin D supplements. In severe symptomatic vitamin D deficiency (i.e., presented with proximal, pelvic-girdle myopathy), using a higher dose of D_3_ repletion, as mentioned in Table 1 or Table 2, patients usually regain their normal daily activities of daily living, like walking and taking care of themselves, within two to three weeks.

While there are protocols where the needed vitamin D doses could be calculated based on body weight [5], none is designed to attain or maintain serum 25(OH)D concentrations at therapeutic levels above 50 ng/mL. When the serum 25(OH)D concentration is unknown, healthcare workers can conveniently use the practical guidance provided in Table 2: a reliable way to replenish tissue stores and maintain serum 25(OH)D concentrations above the minimum therapeutic concentration to have clinical benefits from vitamin D.

For average, non-obese healthy persons, weight-based doses are calculated as 70 to 90 IU/mL/kg/day. For individuals with obesity, 90 to 130 IU/kg/day, and for morbid obesity, 140 to 180 IU/kg/day. In extreme obesity, it can be increased up to 200 IU/kg/day. Information provided in Table 1 (if serum 25(OH)D concentration is known) or Table 2 (using the body weight ratios, when 25(OH)D is unknown) can use for initiation and longer-term maintenance of serum 25(OH)D concentrations [4,45,141].

**Table 2:** Longer-term maintenance schedules of oral vitamin D based on body weight to maintain the levels above 50 ng/mL (125 nmol/L) when the serum 25(OH)D concentrations are unknown.

Bodyweight Category		Dose kg/Day (IU)	Dose (IU) (Daily or Weekly) *	
(Age) or Using BMI (for age > 18) (kg/Ht. M^2^)	Average Body Weight (kg)		Daily Dose (IU)	Once a Week (IU)
(Age 1–5)	5–13	70	350–900	3000–5000
(Age 6–12)	14–40	70	1000–2800	7000–28,000
(Age 13–18)	40–50	70	2800–3500	20,000–25,000
BMI ≤ 19	50–60 (under-weight adult)	60 to 80	3500–5000	25,000–35,000
BMI < 29	70–90 (normal: non-obese)	70 to 90	5000–8000	35,000–50,000
BMI 30–39	90–120 (obese persons) ^#^	90 to 130	8000–15,000	50,000–100,000
BMI ≥ 40 ^$^	130–170 (morbidly obese) ^$^	140 to 180	18,000–30,000	125,000–200,000

* Example of a daily or once-a-week dose range for adults with specific body types (based on BMI for white Caucasians and body weight for other ethnic groups). Appropriate dose reductions are necessary for children. ^#^ For those with chronic comorbid conditions, such as hypertension, diabetes, asthma, COPD, CKD, depression, and osteoporosis, and to reduce all-cause mortality, higher doses of vitamin D are needed. For them, one can use the doses that are recommended for persons with obesity (BMI, 30–39: the third row). ^$^ Those with multiple sclerosis, cancer, migraine headaches, and psoriasis, and those routinely taking medications such as anti-epileptic and anti-retroviral agents that significantly increase the catabolism of vitamin D should consider taking age-appropriate doses recommended for those with morbid obesity (BMI ≥ 40; the higher end of the daily doses in the fourth row).

Using the upfront loading doses of vitamin D_3_ described in Table 1 and Table 2, advances the beneficial clinical effects within days rather than waiting for several months with standard daily or weekly doses. Doubling the daily recommended dose (e.g., to 10,000 IU) for a couple of months and then reducing it to the standard dose reduces the time to achieve the therapeutic target in the circulation to a few weeks.

Recommendation #37 of the 2018-ESPEN Guidelines stated that 500,000 UI vitamin D_3_ could be administered safely as a single dose without any adverse effects [142]. The recommended larger bolus or loading doses of vitamin D, between 100,000 to 400,000 IU, accelerate the intended benefits within three to five days [142]. Many medium-term RCTs confirmed significantly better clinical outcomes from using vitamin D_3_ 50,000 IU capsules, administered (based on the clinical requirement), either once or multiple times a week or once in two weeks, including prevention of symptomatic SARS-CoV-2 [28,134,135,136,137,138,139].

### 5.4. Importance of Parental Vitamin D and 25(OH)D for Generating Calcitriol in Target Cells

While the text above focused on calcifediol and calcitriol as therapies for health and disease, there is evidence of the importance of the parental form of vitamin D in human biology [11]. However, less frequent administration (e.g., intervals of more than once in two weeks) and intermittent high doses or repeated bolus dosing should be avoided in routine clinical activity and RCTs, which is not beneficial [58].

Most steroid hormones enter cells via diffusion and endocytosis as in the kidney and parathyroid gland, via the megalin–cubilin system described above: and in muscle and fat cells [11,143]. Evolutionary, this active cellular entry mechanism for D_3_ and 25(OH)D is set up to prioritise supplying the renal tubules and parathyroid glands for vitamin D’s endocrine functions, even when serum concentrations are low (e.g., 20 ng/mL). Thus, able to generate the hormonal form of calcitriol that is required for the calcium metabolism and musculoskeletal system [12,144].

Many other peripheral target cells do not have the megalin–cubilin system. Therefore, they are dependent on a concentration-dependent gradient for diffusions of vitamin D and 25(OH)D (both free and loosely bound to VDBP and albumin) and, in some instances, endocytosis [circulating D_3_ and 25(OH)D bound to VDBP] into peripheral target cells [9]. Based on the diffusion constants, as illustrated in Figure 1, 25(OH)D is more tightly bound to VDBP than vitamin D [59]. Moreover, the affinity of these molecules to VDBP determines the duration of these compounds in circulation (i.e., the circulatory half-life). These evidence suggest that in normal circumstances, vitamin D is more capable than 25(OH)D of entering target tissues, like immune cells, breast, keratinocytes, brain, and gastrointestinal epithelia. While the circulating concentrations are relatively similar, vitamin D diffuses more freely than 25(OH)D into peripheral target cells from the circulation of 25(OH)D because it is less tightly bound to VDBP.

Therefore, more vitamin D than 25(OH)D expect to enter target cells and become 25-hydroxylated and then 1α-hydroxylated to form calcitriol. If this is the case, the current gold standard, the measured serum concentration of 25(OH)D alone, may not provide the appropriate information or an accurate picture of vitamin D status. Consequently, serum 25(OH)D concentration alone is unlikely to provide complete information on vitamin D adequacy and the requirements for extra-skeletal target cell functions (Figure 1). This highlights the importance of administering vitamin D (right quantities and frequency to maintain steady levels and for persistent effects). The use of calcifediol (for rapid action) in emergencies is discussed in the next section.

### 5.5. Recommended Calcifediol Doses to Boost Serum 25(OH)D Concentration and Immunity against COVID-19

For robust innate and adaptive immune functions, immune cells need to be stimulated by intracellularly generated calcitriol. This initiates autocrine/intracrine and paracrine signalling processes crucial to suppressing inflammatory cytokines and excessive tissue oxidative processes [145,146,147,148] (Figure 5). Administration of calcifediol as a single dose with a high dose of vitamin D or on its own as a weekly dose significantly reduces the severity of acute respiratory distress syndrome (ARDS) in persons with COVID-19 and other infections [78,79].

Several recent systematic reviews conclude that, as with parental vitamin D, early administration of calcifediol reported a significant reduction of complications, intensive care unit admissions, and mortality from COVID-19 [149,150]. A single dose of calcifediol can raise 25(OH)D concentrations into the therapeutic range within four hours [151] without negative consequences [33]. Therefore, in emergencies, such as those present with COVID-19 infection, it is an ideal approach. The recommended body weight-based single, oral calcifediol dose, 0.014 mg/kg (Table 3), boosts the immune system within a day. If higher doses of vitamin D are unavailable, the same dose of calcifediol can be repeated weekly.

If calcifediol is unavailable, cumulative doses of vitamin D_3_ can be substituted, as illustrated in the last column in Table 3. The concomitant administration of calcifediol with a high (bolus) dose of vitamin D, as described in Table 1 and Table 2, not only maintains the serum 25(OH)D concentration at the therapeutic level but also covers during an acute illness and its recovery, for several weeks. This regimen is economical for raising serum 25(OH)D concentration to reach rapidly above 50 ng/mL without measuring serum 25(OH)D concentration. Working with an informed physician with experience is recommended.

## 6. The Rationale for Using Bolus Doses of Vitamin D_3_ and Calcifediol in Emergencies

Oral calcifediol is readily absorbed in the upper gastrointestinal tract and is one-third better absorbed than parental D_3_ [64,102]. With double hydroxylation, calcifediol has a better solubility and absorption profile than vitamin D. It also bypasses the critical the rate-limiting step [144], 25-hydroxylation via CYP2R1 in the liver. Therefore, in contrast to oral D_3_, where patients have to wait for a few days, calcifediol increases serum 25(OH)D concentration within hours [62,63,151].

When the potency of calcifediol is calculated on a weight-to-weight basis for raising serum 25(OH)D concentrations (i.e., bioavailability), it is between 3.2 and 4 times more effective than D_3_ [151]. To simplify the calculation, it is reasonable to take calcifediol as four times more potent than D_3_. For example, when administered as 25 µg/day, an equivalent of 1 µg of D_3_ increased serum 25(OH)D by 1.5 ± 0.9 nmol/L, while one µg of calcifediol increased 25(OH)D by 4.8 ± 1.2 nmol/L [151].

### There Is No Rationale for Using Calcifediol Analogues

There are no meaningful physiological or pharmacological differences between calcifediol and its extended-release analogues. For example, while ordinary calcifediol raises serum 25(OH)D concentrations in four hours, extended-release formulations do the same in three hours [151,152]. With a significantly higher cost of analogues/formulations (parallel with 1α-analogues of calcitriol), there is no advantage of using these instead of calcifediol. The rationale for introducing these synthetic analogues was to differentiate them from the original molecule, calcifediol, merely to obtain protective patents for marketing purposes.

Pharmacodynamic studies reported that calcifediol’s circulatory half-life is between 12 and 21 days [151]. Therefore, it can be administered weekly for a few specific indications, such as hepatic failure, following Roux-en-Y gastric bypass surgery [103], and morbid obesity [119], where medium- or long-term administration of calcifediol is indicated [151]. In the absence of the above situation, administering repeated doses of calcifediol or its analogues as a vitamin D supplement (including for chronic renal failure) to maintain serum 25(OH)D is unwarranted and cost-prohibitive.

Most synthetic vitamin D analogues do not subject to typical physiological feedback control as original vitamin D compounds. Consequently, they have a significantly higher risk of adverse effects [144]. Based on physiology, adverse effects, and the cost, there is no rationale for using calcifediol or its expensive analogues as routine vitamin D supplementation or in renal failure for hypovitaminosis D. Those with advanced renal failure (with normal liver functions) needed parental D_3_ and calcitriol (or its analogues), not calcifediol or its analogues.

For many vitamin D deficient persons with sepsis [10] or COVID-19 [21,22,23,60] (i.e., in emergencies) encountered in clinics or admitted to hospitals, it is unlikely to have information available on their serum 25(OH)D concentrations. For them, administering a body-weight-based proper dose of vitamin D (Tabel 1) or a single dose of 0.014 mg/kg body weight calcifediol (Table 3) can be life-saving. This is approximate 1.0 mg in a non-obese 70 kg adult and 0.5 mg for an adolescent. Such doses are sufficient to rapidly raise serum 25(OH)D to the needed therapeutic concentration (Table 3). Those who are obese need a higher (twice) dose.

## 7. Conclusions

A robust immune system is essential to overcome infections without complications. It depends on the adequate entry of vitamin D_3_ and 25(OH)D into immune cells for generating calcitriol. The latter required maintaining a serum 25(OH)D concentration of over 50 ng/mL. Therefore, to successfully manage and overcome an infectious epidemic or a pandemic, it is crucial to maintain the population’s serum 25(OH)D concentration above the mentioned therapeutic level.

In acutely ill persons, especially those with vitamin D deficiency having infections, raising serum D_3_ and 25(OH)D concentrations quickly is paramount and life-saving. In these urgent situations, 0.5 to 1.0 mg of calcifediol can raise serum 25(OH)D concentrations above the minimum therapeutic levels of 50 ng/mL in four hours and boosts the immune system within a day that facilitates to overcome infections.

While calcifediol raises serum 25(OH)D within hours, the oral administration of even high doses of vitamin D takes three to five days to raise serum 25(OH)D concentrations. This delay is due to its less efficient absorption than calcifediol and the need for vitamin D to undergo 25-hydroxylation in the liver, a rate-limiting step. In acutely ill patients, as in those in the ICU, administering even high doses of oral D_3_ may take a week to increase serum 25(OH)D concentration. Therefore, it is unhelpful in emergencies like SARS-CoV-2 infections.

With a weight-based, single dose of calcifediol, as described in Table 3, circulatory 25(OH)D concentrations are maintained for approximately 8 to 14 days. In contrast, parental high dose vitamin D_3_, administered as loading or bolus, will maintain serum 25(OH)D concentrations between two to three months. Although the circulatory half-life of D_3_ is short, due to the larger initial doses, it maintains a higher circulatory concentration of both D_3_ and 25(OH)D for several weeks—partly because of the release from the storage in fat and muscle tissues.

Therefore, with calcifediol, one should administer a suitable higher dose of vitamin D_3_. This can be done using 50,000 IU vitamin D capsules in outpatients’ setups and emergencies, as illustrated in Table 3. Nevertheless, considering the non-genomic beneficial actions of vitamin D_3_ and its longer duration of physiological actions described above, the combination of D_3_ and calcifediol provides better clinical outcomes than either alone. Therefore, administering the proper doses of D_3_ and calcifediol is recommended for patients with infections as an adjunct therapy at the first outpatient or inpatient encounter.

Multiple observational and RCTs have demonstrated that serum 25(OH)D concentrations (pre-infection or on admission) inversely correlated with the incidence, severity, and rates of death from COVID-19 [45,55,56,153]. Meanwhile, vitamin D supplementation significantly reduces complications and deaths [33,44,45,150,154]. Irrespective of the regimen, initial bolus or loading doses of vitamin D and/or calcifediol should follow a daily or weekly, longer-term maintenance regimen [11,118,155].

The described schedules in the three tables are highly cost-effective ways to raise serum 25(OH)D concentrations and maintain it to keep the immune system on high alert. Consequently, it prevents and/or reduces infections and complications from COVID-19 and other infections. For non-obese 70 kg adults, the recommended longer-term vitamin D_3_ maintenance dose is 5000 IU/(0.125 µg) day or 50,000 IU (1.25 mg)/week (or every ten days). Nevertheless, this regimen takes a few months to reach the desired serum 25(OH)D concentration above 50 ng/mL. It can be expedited by ingesting vitamin D, 10,000 IU/day (250 µg/day) for 8 to 10 weeks and reverting to the daily dose of 5000 IU.

Rectifying vitamin D deficiency costs less than 0.1% of the costs related to evaluating and treating comorbidities and complications associated with vitamin D deficiency [156]. For example, in western countries, vitamin D supplementation to maintain serum 25(OH)D costs approximately $8 per person/year, versus an average cost of $5000 to $15,000/year per person to manage vitamin D deficiency-associated diseases and related complications [156]. Despite a favourable cost-benefit ratio, availability as a non-prescription over-the-counter nutrient, and exemplary safety profile, millions of people become ill due to vitamin D deficiency requiring medical attention, markedly increasing the cost of healthcare. Vitamin D deficiency increases healthcare costs, absenteeism and opportunity costs and reduces productivity.

Considering the described significant benefits associated with disease prevention, reduced illness severity, reduced absenteeism, complications and deaths, improved well-being and higher productivity, the calculated overall cost-benefit ratio for administered vitamin D_3_ supplements exceeds 1 in 20,000. Despite these data, no country is yet to recommend vitamin D (or has published proper guidelines with the right doses) for disease prevention or recommended it as an adjunct therapy to prevent complications and deaths from infections or other diseases. This report provides rationale, justifications, straightforward guidance, and practical tables that provide regimens for use in clinical practice for achieving and maintaining the serum 25(OH)D concentrations needed to ensure a robust immune system that helps to overcome infections, including SARS-CoV-2.

## Figures and Tables

**Table 3:** Using a regimen of calcifediol * to rapidly raise serum 25(OH)D concentration above 50 ng/mL (125 nmol/L) in medical emergencies (i.e., to raise serum levels within four hours). ** A single body weight based, oral dose is calculated: 0.014 mg/kg body weight.

Weight (lbs)	Weight (kg)	Calcifediol ~ (mg) ^#^	If Calcifediol Is Not Available: Bolus/Loading Dose of Vitamin D_3_ ^##^
8–14	4–6	0.05	20,000
15–21	7–10	0.1	40,000
22–30	10–14	0.15	60,000
31–40	15–18	0.2	80,000
41–50	19–23	0.3	100,000
51–60	24–27	0.4	150,000
61–70	28–32	0.5	200,000
71–85	33–39	0.6	240,000
86–100	40–45	0.7	280,000
101–150	46–68	0.8	320,000
151–200	69–90	1.0	400,000
201–300	91–136	1.5	600,000
>300	>137	2.0	800,000

* Calcifediol [partially activated vitamin D_3_, 25(OH)D]. ** Use the earliest possible in person with COVID-19, sepsis, Kawasaki disease, multisystem inflammatory syndrome, acute respiratory distress syndrome, burns, and vitamin D deficiency in early pregnancy and other clinical emergencies. ^#^ Measurement (or the concentration) of serum 25(OH)D is unnecessary. ^##^ If calcifediol is unavailable, the equivalent dose of vitamin D is administered, as illustrated in Table 2, preferably in divided doses over three to five days. Irrespective of the regimen used, daily or weekly follow-up maintenance vitamin D dose is necessary as described in the text.

## Data Availability

Not applicable.
